# Patient experience with non-invasive prenatal testing (NIPT) as a primary screen for aneuploidy in the Netherlands

**DOI:** 10.1186/s12884-022-05110-2

**Published:** 2022-10-20

**Authors:** Syanni A. Kristalijn, Karen White, Deanna Eerbeek, Emilia Kostenko, Francesca Romana Grati, Caterina M. Bilardo

**Affiliations:** 1 Medical Affairs, Roche Diagnostics Netherlands, Almere, The Netherlands; 2 Roche Diagnostics, San Jose, CA USA; 3 Management Policy Analysis and Entrepreneurship, VU University Amsterdam, Amsterdam, The Netherlands; 4 TOMA Advanced Biomedical Assays Laboratory, Busto Arsizio, Varese Italy; 5 Amsterdam University Medical Center, Amsterdam, The Netherlands

**Keywords:** Non-invasive prenatal testing, Prenatal screening, Genetic counseling, Patient experience, Genome-wide

## Abstract

**Background:**

Non-invasive prenatal testing (NIPT) as a screening method for trisomy 21 and other chromosomal abnormalities has been adopted widely across the globe. However, while many clinical validation studies have been performed, less is known regarding the patient experience with NIPT. This study explored how individuals experience NIPT in a pre- and post-test setting, where NIPT is broadly available as a primary screening method with the option of reporting beyond common trisomies.

**Methods:**

Participants were recruited using social media with a strategy designed to select individuals who had the option to have NIPT as part of the TRIDENT-2 study (In the Netherlands, NIPT is only available within the TRIDENT studies executed by the NIPT consortium. This research was done independently from the NIPT consortium.) in the Netherlands. The study used online questionnaires and semi-structured interviews. Both were developed around a patient experience framework consisting of seven themes: information, patient as active participant, responsiveness of services, lived experience, continuity of care and relationships, communication, and support.

**Results:**

Overall, 4539 questionnaire responses were analyzed and 60% of the respondents had experienced NIPT. Of those, 1.7% received a high-risk result for trisomy or another chromosomal copy number variant (referred to as an “additional finding”). Overall, participants felt they had received sufficient information and had control over their decision regarding whether or not to choose NIPT. The vast majority of respondents who had NIPT were positive about their experience and would use it again. Those with results showing an increased probability for trisomy or additional findings were more likely to report negative feelings such as tension and anxiety, and less likely to feel that they had been sufficiently prepared for the implications of their results.

**Conclusions:**

The patient experience with first-tier NIPT in the Netherlands was largely positive. Areas for improvement included counseling on the implications of screening and the different possible outcomes of NIPT, including additional findings that may be uncovered by expanding NIPT beyond the common trisomies. The experiences reported in this study may be useful for other countries intending to implement NIPT.

## Background

Non-invasive prenatal testing (NIPT) using cell-free DNA became clinically available in 2011 and has been shown to be a highly accurate method to screen for fetal trisomy 21 [1]. Over the last decade, NIPT test offerings have expanded beyond screening for trisomy 21 to include screening for trisomy 18, trisomy 13 and sex chromosome aneuploidy. More recently, there has been increasing use of “genome-wide” NIPT, which includes the reporting of other chromosomal copy number variations, usually limited to a resolution of 5–7 Mb or greater [2]. The use of genome-wide NIPT has been the subject of some controversy because of concerns that it increases false positive rates and, to date, has less evidence supporting performance and clinical utility [3–5]. Furthermore, it increases the likelihood of findings with uncertain clinical significance, which may lead to patient uncertainty and anxiety [6, 7].

NIPT has been adopted widely across the globe but with great variation in its implementation and reimbursement [8]. In two European countries (Belgium and the Netherlands), NIPT is offered as a primary screening test in all pregnancies (“first-tier” screening). In other countries or regions with reimbursement by public healthcare systems, NIPT is offered as a second tier test in high-risk pregnancies after other first trimester screening tests, mainly the combined test. In the Netherlands, NIPT is available as a first-tier screen as part of the TRIDENT-2 study [9]. Certified counselors, most of whom are primary care midwives, provide pre-test counseling in distinct 30-minute sessions. Individuals opting for NIPT can choose screening for trisomy 21, trisomy 18, and trisomy 13 with or without the reporting of other copy number variants (referred to as “additional findings”). Sex chromosomes are not investigated. Post-test counseling is provided by obstetricians in the case of results positive for the common trisomies and by geneticists for results positive for additional findings. The out-of-pocket cost for NIPT is 175 euro, regardless of the type of testing chosen. During the first year of the TRIDENT-2 study, 78% of women choosing NIPT opted for the genome-wide test [9].

Understanding the patient experience is increasingly important across all healthcare settings [10]. While there is an extensive body of published clinical evidence for NIPT validation and clinical utility, less research has been carried out on the patient experience. Existing research has been carried out mostly in high-risk populations and often with a particular focus on informed choice [11–14]. To date, few studies have been performed in populations with first-tier screening models or with genome-wide screening [15–17]. The TRIDENT-2 study is collecting data on women’s perspectives, but this is yet to be published.

The goal of this study was to explore how patients experience the entire NIPT process, from pre-test counseling to the communication and management of results, in a setting where NIPT is available as a first-tier screen with the option of reporting beyond the common trisomies. A secondary goal was to explore how the patient experience varied according to the test result.

## Methods

### Study design

The study used a mixed method approach, consisting of an online questionnaire and semi-structured interviews.

### Participants

Participants were recruited using an advertisement on Facebook (Palo Alto, CA, USA) that was targeted to pregnant women and women with young children in the Netherlands. A link from the advertisement led to an online questionnaire administered by SurveyMonkey Inc. (San Mateo, CA, USA). The advertisement and questionnaire were written in Dutch. Individuals were asked to complete the questionnaire if they had a pregnancy after April 2017 or were currently pregnant with at least 20 weeks’ gestation, after giving their consent. This design was intended to recruit participants who would have had the option of NIPT under the TRIDENT-2 study and, if currently pregnant, had completed the counseling and testing process. Responses were gathered between May 15 and May 31, 2020.

After completing the questionnaire, individuals were asked if they would be willing to participate in an interview. If so, they were asked to provide an email address in a separate form that was not linked to the questionnaire responses. From the pool of respondents, interviewees were selected based on three main criteria. From the pool of respondents, interviewees with positive results (or other unique situations) were selected in proportions such that their choice of NIPT (trisomy only vs. reporting additional findings) and results (positive for trisomy vs. an additional finding) reflected the study population overall. Individuals with multiple pregnancies (and potentially different decisions) were prioritized.

### Questionnaire

The first section of the questionnaire ascertained patient age, education, decision about NIPT (yes or no), choice of NIPT (trisomy-only or reporting additional findings), the results of NIPT and the results of diagnostic testing, if applicable. In the questionnaire and also in this manuscript, results showing an increased probability of trisomy or additional findings were termed “positive” and results showing low probability were termed “negative.” Although the survey was explicitly targeted to pregnant or previously pregnant women, the questionnaire did not request information about the sex or gender of the participant.

The second section of the questionnaire consisted of a series of questions designed to capture the patient experience throughout the counseling and testing process. The structure of the questionnaire was based on the Warwick Patient Experiences Framework [18]. Table 1 provides a brief summary of the framework and the focus of the questions in this study. Some questions cascaded to additional questions, meaning that all questions were not asked of every participant. Participants could also proceed through the questionnaire without being required to provide an answer to each question. Most questions used five-point Likert scales with possible answers being: strongly disagree, disagree, neutral, agree, and strongly agree. The questionnaire is available in the supplementary information [see Additional file 1 (original in Dutch) and 4 (translated to English)].

**Table 1:** The Warwick Patient Experiences Framework

Theme	Description and focus in this study.
Information	Information enables active decision making. Includes sources and quality of information.
Patient as active participant	Patients play an active role in their healthcare. This is associated with outside influences and control.
Responsiveness of services – an individualized approach	The patient is seen as an individual in the healthcare system. Includes how well services are performed and needs are met.
Lived experience	The recognition that individuals experience their condition in a unique way. Expectations and feelings are key themes.
Continuity of care and relationships	Relates to accessibility and barriers to services. Includes trust and recognition of the expertise of the healthcare professional.
Communication	The setting and skills of the healthcare professional facilitate two-way communication. Involves enabling questions and providing answers.
Support	Patients have preferences for support (emotional and practical). Responsiveness of professionals to support individual needs.

### Interview

The interviews took place by video call or telephone call between May 27, 2020 and June 5, 2020. They followed a guide [see Additional file 2], which was structured similarly to the questionnaire, and were designed to last 30–45 minutes. The interviews were transcribed and coded by DE and the transcriptions and summaries shared with the interviewees. KW and SK performed the translations from the questions and quotes. Differences were solved by consensus or consulting a third investigator (KB).

### Data analysis

Survey responses were excluded if: the participant did not indicate whether or not they had NIPT; less than 1 min was spent to complete the full questionnaire; or answers were given to fewer than 20 questions. Because the survey allowed only one response per browser, each response submitted was considered to be from a unique participant.

For analysis of the questionnaire, responses to the five-point Likert scale questions were converted to three categories: disagree (strongly disagree or disagree), neutral, and agree (strongly agree or agree). For individual questions, the percent of respondents who agreed or disagreed was calculated as a proportion of all responses (agree, disagree, and neutral). Comparisons between groups only considered the respondents agreeing vs. disagreeing and used a two-tailed Fisher Exact Test to determine significance.

All interviews were recorded and transcribed. The transcripts were coded based on the Warwick framework and analyzed with ATLAS.ti 8.4.24 (ATLAS.ti Scientific Software Development GmbH, Berlin, Germany).

All methods were performed in accordance with the relevant guidelines and regulations.

## Results

During the 16 days of recruitment, 4979 questionnaire responses were received. After excluding 440 responses based on the criteria described above, 4539 questionnaires were available for analysis. Table 2 provides the respondents’ demographics and NIPT uptake. Of the 2719 individuals (59.9%) who had NIPT, 2637 (97%) reported having negative results; 46 (1.7%) reported having positive results; 36 (1.3%) reported receiving no results (Table 3). Diagnostic information was disclosed by 41 of the respondents with positive results. In total, 20/26 trisomies (76.9%) and 7/15 additional findings (46.6%) were confirmed. No further information was obtained on the cases in which the screening test did not yield a result.

**Table 2:** Characteristics of all participants

	N	%
Age		
< 25	163	3.6
25–29	1386	30.5
30–34	1970	43.4
≥35	1020	22.5
Educational level^a^		
Low	43	0.9
Intermediate	1670	36.8
High	2808	61.9
Declined to state	18	0.4
NIPT uptake		
Did not have NIPT	1820	40.1
Had NIPT	2719	59.9
Trisomy only	647	14.3
With additional findings	2072	45.6

^a^Low: primary education, primary vocational education (lbo/vmbo); Intermediate: middle secondary education (mavo), middle vocational education (mbo), higher secondary education (havo), pre-university education (vwo); High: university education (wo), higher vocational education (hbo)

**Table 3:** NIPT results

	N	%
NIPT for trisomy only		
Negative	629	97.2
Positive trisomy	8	1.2
No result	10	1.5
NIPT with additional findings		
Negative	2008	96.9
Positive trisomy	18	0.9
Positive additional finding	20	1.0
No result	26	1.3

*NIPT* Non-invasive prenatal testing

Trisomy refers to trisomy 21, 18 or 13. Negative: results showing low probability of trisomy or other copy number variation (additional findings); positive: results showing increased probability of trisomy or other copy number variation (additional findings)

Of the 47 respondents willing to be interviewed, 21 responded on the request for additional information on their pregnancies. Eight candidates (6 with positive results and 2 with negative results but with subsequently identified fetal anomalies) were selected based on their unique experience with NIPT in one or more pregnancies using the criteria described in Table 4.

**Table 4:** NIPT choices and results in pregnancies of interviewed participants

Identifier	Pregnancy number	NIPT choice	NIPT result	Clinical outcome
R1	1	With additional findings	Positive for trisomy 21	Confirmed (amniocentesis)
	2	Trisomy only	Negative	N/A
R2	1	Trisomy only	Positive for trisomy 21	Confirmed (CVS)
R3	1	With additional findings	Negative	N/A
	2	With additional findings	Positive for trisomy 21	Confirmed (CVS)
	3	With additional findings	Negative	N/A
R4	1	With additional findings	Negative	Renal abnormality^a^
R5	1	With additional findings	Positive for additional finding	Maternal variant
R6	1	Trisomy only	Negative	Chromosome abnormality not detectable by NIPT^a^
	2	No NIPT	N/A	N/A
R7	1	With additional findings	Positive for additional finding	Inconclusive CVS; apparently normal phenotype
R8	1	With additional findings	Positive for additional finding	Maternal variant

*CVS* Chorionic villus sampling, *N/A* Not applicable, *NIPT* Non-invasive prenatal testing

Trisomy only refers to trisomy 21, 18 or 13. Negative: results showing low probability of trisomy or other copy number variation (additional findings); positive: results showing increased probability of trisomy or other copy number variation (additional findings).

^a^Identified later in pregnancy

### Information

**Fig. 1:**
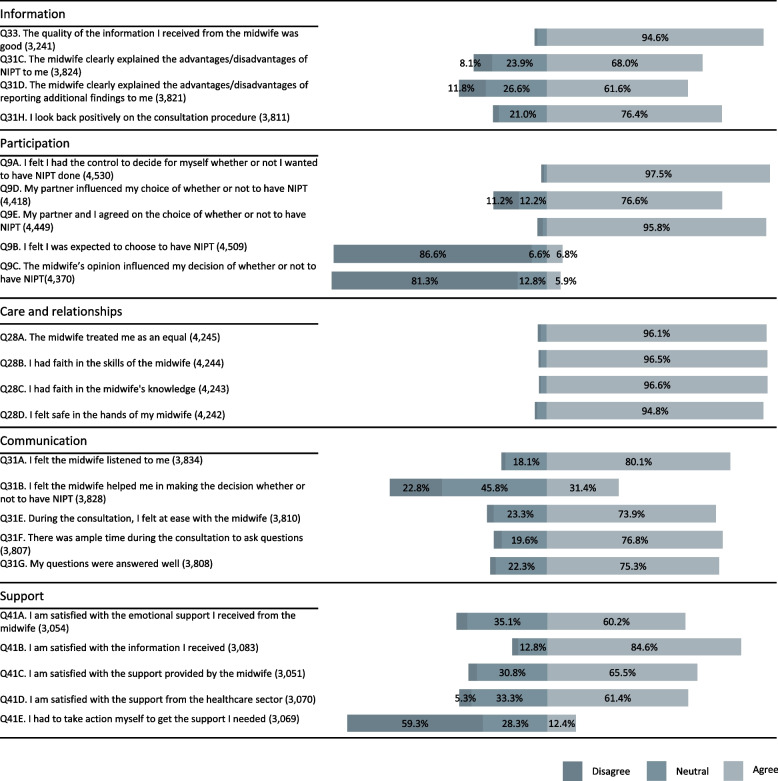
Responses of the entire cohort. The number of individuals responding to each statement is given in parentheses. The bars represent the proportion of individuals disagreeing, neutral, and agreeing. NIPT, non-invasive prenatal testing

The majority of participants (4181/4337; 96.4%) received information about NIPT from a midwife. Of these, 94.6% felt they had received good quality information but only 68.0% felt that the advantages and disadvantages of testing were clearly explained (Fig. 1). In addition, 2814 (64.9%), 2557 (59.0%), and 774 (17.8%) individuals reported receiving information from the information brochure, the internet, and family and friends, respectively.

**Fig. 2:**
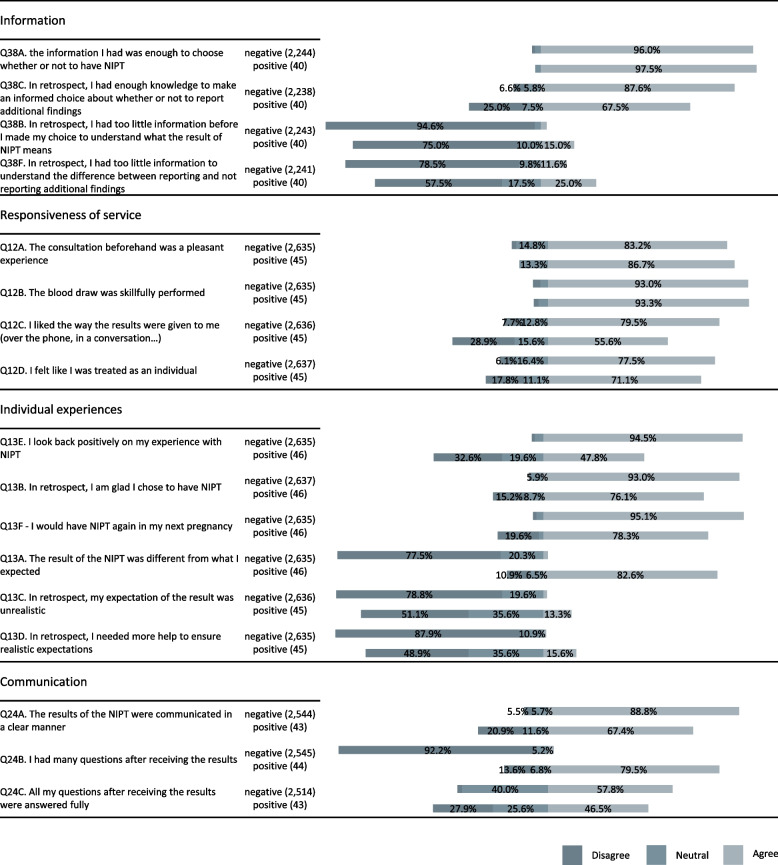
Responses of individuals who had NIPT. Subgroups that had negative results and positive results are plotted separately for comparison. The number of individuals responding to each statement is given in parentheses. The bars represent the proportion of individuals disagreeing, neutral and agreeing) NIPT, non-invasive prenatal testing

Irrespective of whether they had positive or negative screening results, 95% of the respondents who had NIPT felt they had sufficient information to make an informed choice (Fig. 2). However, 15% of those with positive results agreed that, in retrospect, the information they had received was insufficient to understand what a NIPT result really meant. In hindsight, 25% of those with positive results felt that they had not made an informed choice about reporting or not reporting additional findings.

In addition, 5 of 6 interview participants reported not having been sufficiently informed about the meaning of a positive result. One interviewee, for example, reported that they were not aware of the possibility that the NIPT result might not be confirmed. Another, with a result positive for additional findings, said they had chosen this option to know “everything,” without realizing it could lead to more uncertainty. The two interviewees with negative NIPT results felt they were falsely reassured by the test when they were confronted with abnormal ultrasound findings later in pregnancy.

### Participation – the patient as an active participant

97.5% of participants felt they had control over the decision to have NIPT (Fig. 1). A small percentage felt external pressure: 6.8% felt that they were expected to choose NIPT and 5.9% felt that the midwife influenced their decision. One interview participant stated the following:

*So it was really like “actually you just have to do it, because then you know, and that’s good for you” so to speak. Actually you have to do it, but of course it is your own choice.* [R4].

Compared to those that had NIPT, respondents that did not have NIPT were less likely to feel they had control over their decision (*p* < 0.5), more likely to feel they were expected to have NIPT (p < 0.5) and more likely to feel influenced by the midwife (*p* < 0.5).

### Responsiveness of services – an individualized approach

The majority of survey participants who had NIPT were positive about the service they received during the screening process (Fig. 2). 7.7% of women with negative results and 28.9% with positive results did not like the way the results were communicated to them. Interview participants described being called at work or in the car and one had difficulty in reaching the right department after a missed call.

### Individual experiences – lived experience

Figure 2 shows the experiences and expectations of survey participants who had NIPT. Overall, more than 90% looked back positively on their experience, were glad about their choice, and would choose NIPT again in another pregnancy.

In the group with positive results, 76% were glad they chose NIPT and 78% would do so again. However, 83% reported that the screening result was different from their expectations and 16% agreed that, in retrospect, they would have needed more help to ensure realistic expectations. Among the 24% of individuals with positive results who regretted having NIPT and would not choose it again, 5/7 and 7/9, respectively, had false positive results.

**Fig. 3:**
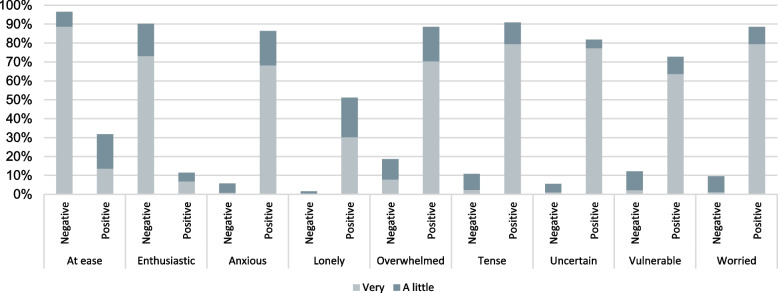
Responses of individuals having NIPT about their feelings after receiving the results. Subgroups that had negative results and positive results are plotted separately for comparison. The bars represent the proportion of individuals experiencing a lot (medium gray) or a little (light gray) of each emotion. NIPT, non-invasive prenatal testing

When asked about feelings experienced after receiving NIPT results (Fig. 3), the majority of those with negative results felt relieved and reassured; however, almost 20% reported being at least a little overwhelmed. Not surprisingly, participants receiving positive results reported tension, worry, anxiety, uncertainty and vulnerability.

### Care and relationships – continuity of care and relationships

The vast majority (95%) of survey participants who received information about NIPT from a midwife felt the midwife treated them as an equal and had faith in the midwife’s skills and knowledge (Fig. 1). There was no difference in responses between the groups having or not having NIPT.

About one fifth (19.3%; 775/4027) of respondents agreed that cost was a major factor in their decision about NIPT. Not surprisingly, the proportion was higher in the group that did not have NIPT than in the group that did (*p* < 0.05).

### Communication

There was general satisfaction with the midwives’ communications during counseling (Fig. 1). However, 23% felt that the midwives had not helped them in their decision to have NIPT or not.

The majority of respondents felt that the NIPT results were communicated clearly (Fig. 2). The proportion of individuals disagreeing with this was higher in the group with positive NIPT results (*p* < 0.05). Overall, 80% of the group reported having many questions and 28% did not feel their questions were fully answered.

### Support

Respondents to the survey cited family and friends (1542) and information given by the midwife (1462) as sources of support throughout the NIPT process.

Over 90% of respondents were neutral or agreed to statements expressing satisfaction with the support they received from the midwife and the healthcare sector (Fig. 1). In total, 16% of those with positive results were dissatisfied with the support provided by the midwife and 31% felt they needed to take action to find additional support.

Interview participants had different experiences in the support they received after a positive result:

*Well, I experienced that my midwifery practice was very involved. [ … ] I hope all midwives do, but I was very pleased that they sympathized.* [R5]

*Sure you get leaflets, and that's just very clinical. But yes, you are not prepared for that emotionally. And what it does to your relationship, you are not prepared for that either. I mean I yelled that we needed help.* [R3]

Two interviewees felt that it would have been helpful to be connected with someone who had been through the process; one of them indicated that they had offered to do this for others.

### Experiences with post-test management of positive NIPT results and differences by result type

**Fig. 4:**
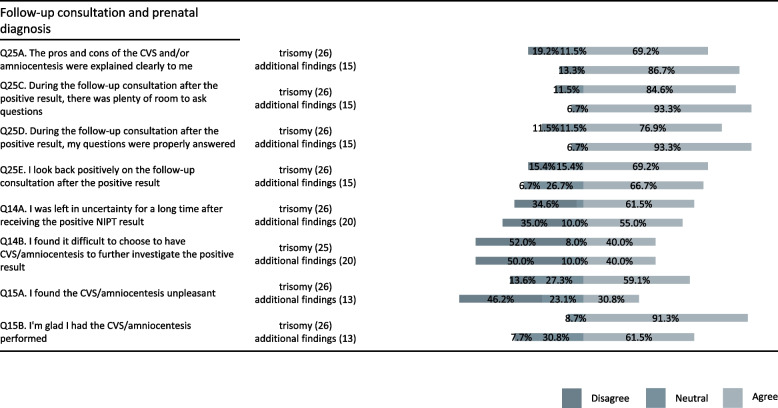
Responses of individuals who had positive NIPT results. Subgroups that had trisomy-positive results and additional findings are plotted separately for comparison. The number of individuals responding to each statement is given in parentheses. The bars represent the proportion of individuals disagreeing, neutral and agreeing. CVS, chorionic villus sampling; NIPT, non-invasive prenatal testing

Overall, 80–90% of respondents were neutral or looked back positively on the consultation, were satisfied with the explanation of the pros and cons of diagnostic testing, felt that there was room to ask questions and felt their questions were answered (Fig. 4). In general, across the questions, the proportion of individuals satisfied with the consultation was higher in the group with additional findings than the trisomy group but none of the differences were statistically significant. One interview participant expressed their satisfaction as follows:

*The follow-up process was completely voluntary and that was clearly emphasized, which was nice [ … ]. The process was clearly explained with all the steps. [R5]*

In both the trisomy-positive and additional finding-positive groups, survey respondents were split as to the extent of uncertainty after receiving the NIPT result, the difficulty in making a decision about prenatal diagnosis, and how unpleasant the diagnostic procedure was. Individuals with results positive for additional findings were less likely than those with trisomy-positive results to be glad they had prenatal diagnosis but the difference was not statistically significant (*p* = 0.3).

Interview participants talked about uncertainty, decision-making, and waiting for results:

*In total it took about a month. [...] You are continuously occupied with it, trying to find more information. [...] It does not stop until you make further decisions, and even then it does not stop.* [R8]

Another interviewee expressed ongoing uncertainty, even after receiving diagnostic results:

*If those three [Down-, Edwards- and Patau Syndrome] would be in order, then it would be good. Therefore, I regret doing the additional findings. Especially because the result came back positive, but we still do not know if the baby has it or not. It is still not clear. He is healthy and he is doing well, but whether this had any influence on the baby, what they found, we still do not know. [R7]*

In the areas of the questionnaire where there was a difference between the responses of the groups with positive and negative results, the responses were further analyzed according to the type of result. Compared to respondents with trisomy-positive results, those with results positive for additional findings were more likely to agree that the results were different from their expectation. They were less likely to be satisfied with the way the results were given and to feel that they had made an informed choice with respect to reporting additional findings. None of these differences were statistically significant. There was a significant difference (*p* < 0.05) in the proportion agreeing vs. disagreeing with the statement “I had to take action myself to get the support I needed.” Overall, 45.8% of respondents with test results positive for trisomy agreed with this statement compared to 6.7% of those with additional findings.

### No-results

There was a similar pattern of response in the group whose NIPT did not yield a result as in the group with positive results. Overall, 20.7% did not feel that they had sufficient knowledge to make a choice about NIPT and 27.6% felt that they had too little information to understand what the result of NIPT means. Although 71.4% felt the results of the NIPT were communicated clearly, 82.9% had many questions and only 31.4% reported their questions were answered.

## Discussion

This study explored multiple aspects of patient experience with the NIPT screening program in the Netherlands. With the use of social media, more than 4500 survey participants were recruited in 16 days. This remarkably high recruitment, without any survey incentives, suggests that individuals have a personal need to share their prenatal screening experiences.

Overall, participants reported that they had sufficient information to make a decision about whether or not to have NIPT and had control over the decision-making. Participants were satisfied with the service and communication, and felt that they had received sufficient support throughout the process. The vast majority of participants having NIPT were positive about their experience and would have NIPT again in a future pregnancy. Not surprisingly, experiences were less favorable in participants with positive results, especially regarding information, individual experience and communication. This group reported more frequently that the results were unexpected, that induced feelings such as anxiety, tension and worry, and that they did not like the way the results were communicated to them. This group also reported that they needed to take action to get the support they required and were less likely to feel sufficiently prepared for the implications of the results. This was especially the case for individuals who had opted for additional findings. A quarter of women with positive results felt they had not made an informed choice about reporting or not reporting additional findings. Most of the interviewees reported not having been sufficiently informed about the limitations of positive or negative results and 16% of women with positive results agreed that, in retrospect, they would have needed more help to ensure realistic expectations.

The responsible implementation of a prenatal screening program relies on protecting each individual’s freedom to choose or decline screening [4]. In this study, almost all respondents felt that it was their decision whether or not to have NIPT. Despite this, 7% felt they were expected to choose NIPT and 6% reported that the midwife influenced their decision. A recent publication from the TRIDENT-2 study showed that a similar proportion of respondents (5% overall) felt societal pressure to accept screening; however, almost none reported accepting or declining because of the midwife or doctor [19]. Together, these studies show that the emphasis on the freedom to choose as one of the key components in the Dutch screening program is effective, but that it needs ongoing protection. This concept will face similar or greater challenges in other countries depending on the economic and cultural context in which NIPT is implemented [20].

Almost 20% of the participants in this study agreed that the cost of NIPT was a factor in their decision about having screening. Previous surveys have also identified the cost of screening as a factor in test uptake [21, 22]. Ethical arguments have considered that a threshold cost might promote informed consent by encouraging more careful decision-making and avoiding routinization (the perception that screening is an ordinary aspect of routine care); however, an overriding concern is that cost is a barrier to equitable access [23]. One study found some evidence for the former when it presented vignettes to Dutch citizens and found that respondents agreed less with a pregnant woman declining NIPT when the testing was fully funded [24]. A recent study showing that NIPT uptake in the Netherlands was two times lower in socioeconomically disadvantaged neighborhoods provides strong support for the latter [25]. The out-of-pocket cost for NIPT will disappear in the Netherlands as of April 2023 [26], which will eliminate the barrier to equitable access, but may introduce other concerns such as less informed decision-making and routinization.

Participants were uniformly positive about their comfort with the midwife and quality of the information received, but it is notable that about one quarter reported that the midwife did not help them in decision-making about NIPT. Although respect for individual autonomy is paramount, individuals may need varying levels of support beyond the provision of information, such as help in clarifying their values, in order to make an informed decision. In a 2019 Dutch survey, Martin et al. [16] established that about half of participants considered decision-making support important or very important but found that decision-making support was weak compared to two other aspects of pre-test counseling (client-counselor relationship and health education). This may require the use of additional tools but is a possible area for improvement in the Netherlands and a consideration in the implementation of programs in other countries.

This study was consistent with studies using both first or second-tier screening paradigms in finding individuals having NIPT to be generally positive about the experience and intending to have NIPT again in a future pregnancy [12, 14, 15, 17, 27]. This study is unique in that it included 46 individuals with positive screening results. Fewer respondents in this subgroup were positive about the experience and likely to choose NIPT in another pregnancy. This appears to be largely due to individuals whose results were not confirmed on diagnostic testing. It does appear that a sizable minority of individuals with positive results felt they had not received sufficient information to understand the implications of the results. This was also the case for some participants with negative results, albeit less often. Those with results positive for additional findings in this study were more likely to report that the results were different from their expectation and less likely to feel that they had received sufficient information to make an informed choice. A recent Australian study of individuals having NIPT (mostly as a first-tier screen) found this to an even greater extent; 94% of the population reported that they had adequate information but only 66% felt they were sufficiently informed of the consequences of a high-risk result [15]. The investigators conclude that pre-test counseling should address the entire process of prenatal screening, including both the medical and psychological consequences of positive results. This may be more challenging when NIPT is expanded to a wider range of conditions because it increases the range of possible outcomes [4]. Investigators interviewing Dutch women about expanded screening remarked that their interviewees found it difficult to understand what type of findings there might be [21].

In the current study, individuals with positive test results for additional findings were less likely to feel positive that a prenatal diagnostic procedure had been performed. The reason for this may be that the chance of a false-positive or inconclusive result is much higher in this group. For one of the interviewees in this study, lack of certainty about the clinical significance of the NIPT result remained even after the diagnostic result was available. This is inherent to the range of possible results and implications of a positive additional finding result [5]. Interestingly, participants with additional findings were more likely to be satisfied with the follow-up consultation and less likely to report that they needed to search for additional support themselves. This might be due to the policy of referring this subset of patients to geneticists for follow-up care. These care providers may have more familiarity with the counseling of results of uncertain significance, with rarer conditions and support and resources available to families.

The strength of this study is the size of the cohort; with 4539 participants, it was large enough to include a full range of experiences i.e., it included individuals who did and did not have NIPT as well as those with a full range of test results. This was due to an extremely efficient recruitment strategy; however, there is potential for bias introduced during recruitment. Survey-based health research generally has lower levels of participation by populations with lower socioeconomic status [28]. Although the use of social media, as was the case here, can reach more diverse populations than traditional clinic-based recruitment [29], most of the respondents in this study were highly educated. Compared to the published TRIDENT-2 data [9], this population also had a greater representation of individuals choosing NIPT (60% vs. 42%) and of individuals with positive results (1.7% vs. 0.84%). Although individuals with positive results may be more likely to take the survey and to share their (negative) experience the number was still relatively low. Also, the disclosure of the sponsor of the study being a commercial entity with a NIPT product could introduce bias in participation despite the privacy policies in place. This may be less of a concern in this study because the sponsor’s product is not available in the Netherlands. A limitation of the study is that, in spite of the relatively high number of respondents with positive results, the study was still underpowered to show significant trends in the comparison between NIPT for trisomies only or with additional findings. Hence, larger studies focusing on this group are necessary to judge upon the true impact of an extended NIPT menu offer on patients’ well-being. Another possible limitation is that the questionnaire did not use established measures to focus precisely on aspects such as informed consent or anxiety and was not formally validated. On the other hand, the more flexible design allowed for exploration of patient experiences across seven different themes. This framework gave the study a broad view of a complex experience.

This study was specific to the Netherlands and, although the findings cannot be generalized to all other countries, many of the themes addressed here are of interest when implementing screening programs regardless of location. As NIPT is introduced as a first-tier screening in increasingly more countries, further study of the patient experience will be valuable to optimize its implementation. A deeper investigation of the experiences of individuals with results positive for additional findings, including those reflecting maternal genetic status or an imminent miscarriage, those that remain confined to placenta or are confirmed in a mosaic form in the fetal tissues, in a larger sample is therefore warranted. It would also be valuable to study the experience of individuals whose tests did not yield a result and of ethnic minorities as these were not in scope of this study.

A last aspect we would like to stress is the importance of offering NIPT in combination with a scan to check fetal anatomy. This should ideally be done prior to the NIPT [30], however, from September 2021 an anatomy scan at 13 weeks, next to NIPT, has been offered in the Netherlands in a research setting. It will be interesting to collect the opinions of women regarding this new screening and to verify the clinical consequences of its interaction with NIPT.

## Conclusions

The results of this study show that patients’ experiences with first-tier NIPT in the Netherlands are largely positive. However, the complexity of this novel test, with all its implications, must be considered in relation to the information that should be provided to women to enable fully informed decision-making. The results of this study stress the importance of providing thorough, non-directive, transparent and standardized information on the medical as well as psychological aspects and implications of the test. This would also include the interventional nature of the study and its practical implications when involving positive results for conditions for which a clinical significance cannot be predicted. A clear training and pre- and post-counseling guide for healthcare professionals should therefore be available to ensure the quality of the counseling and decision-making. When a positive test result is communicated, the healthcare professional needs to take sufficient time to address all questions, including those arising at later time points.

Some findings point to areas for improvement in the counseling for individuals with results positive for additional findings. This counseling should better support informed decision-making and better prepare individuals for the implications of the different range of NIPT results and outcomes, including the possibility that a positive result might be entirely inconsequential. Especially rare autosomal trisomies are likely to be confined to the placenta (mosaicism) or maternal in origin (including malignancies), related to an imminent miscarriage, or remain of uncertain significance even after diagnostic testing.

## Data Availability

The datasets used and/or analyzed during the current study available from the corresponding author on reasonable request**.**

## Abbreviation

NIPT: Non-invasive prenatal testing
